# MicroScope: an integrated platform for the annotation and exploration of microbial gene functions through genomic, pangenomic and metabolic comparative analysis

**DOI:** 10.1093/nar/gkz926

**Published:** 2019-10-24

**Authors:** David Vallenet, Alexandra Calteau, Mathieu Dubois, Paul Amours, Adelme Bazin, Mylène Beuvin, Laura Burlot, Xavier Bussell, Stéphanie Fouteau, Guillaume Gautreau, Aurélie Lajus, Jordan Langlois, Rémi Planel, David Roche, Johan Rollin, Zoe Rouy, Valentin Sabatet, Claudine Médigue

**Affiliations:** 1 LABGeM, Génomique Métabolique, CEA, Genoscope, Institut François Jacob, CNRS, Université d'Évry, Université Paris-Saclay, Evry, 91057, France; 2 UMS 3601 IFB-core, CNRS, INRA, INSERM, CEA & INRIA, Genoscope, Evry, 91057, France

## Abstract

Large-scale genome sequencing and the increasingly massive use of high-throughput approaches produce a vast amount of new information that completely transforms our understanding of thousands of microbial species. However, despite the development of powerful bioinformatics approaches, full interpretation of the content of these genomes remains a difficult task. Launched in 2005, the MicroScope platform (https://www.genoscope.cns.fr/agc/microscope) has been under continuous development and provides analysis for prokaryotic genome projects together with metabolic network reconstruction and post-genomic experiments allowing users to improve the understanding of gene functions. Here we present new improvements of the MicroScope user interface for genome selection, navigation and expert gene annotation. Automatic functional annotation procedures of the platform have also been updated and we added several new tools for the functional annotation of genes and genomic regions. We finally focus on new tools and pipeline developed to perform comparative analyses on hundreds of genomes based on pangenome graphs. To date, MicroScope contains data for >11 800 microbial genomes, part of which are manually curated and maintained by microbiologists (>4500 personal accounts in September 2019). The platform enables collaborative work in a rich comparative genomic context and improves community-based curation efforts.

## INTRODUCTION

The use of high-throughput ‘-omics’ approaches produces a vast amount of information that needs to be efficiently integrated and analyzed to understand the biological meanings encoded in living organisms. Despite the development of efficient bioinformatics approaches, maintaining consistency and accuracy in genome annotation remains a challenging task. Several integrated environments for the analysis of prokaryotic genomes that combine and standardize information from a variety of sources and apply uniform (re-)annotation techniques have been developed (e.g. Ensembl Bacteria (1), IMG (2), PATRIC (3)). However few of them support systematic and efficient revision of the automatic annotation of gene functions, a process that adds great value to resources. At the French National Sequencing Center (CEA/DRF/Genoscope), we have developed the MicroScope platform (https://www.genoscope.cns.fr/agc/microscope) which is an integrated software environment for data management, annotation, comparative analysis and visualization of prokaryotic genomes especially for the review of the quality of functional annotation. Published for the first time in 2006 under the name MaGe (4), the platform has been under continuous development within the LABGeM team at CEA, and its capacities are now quite extensive (5–7).

In summary, MicroScope supports free-of-charge external submissions of assembled genomes and metagenomes (i.e. Metagenome-Assembled Genomes, MAGs) generated by any sequencing technology or collected from public sequence data archives. Submission of reads for RNA-seq and variant analysis is also available for genomes already integrated into MicroScope. All genome submissions are processed through several analysis workflows for the syntactic and functional annotation (a detailed list of software and databases integrated in the MicroScope workflows is given in (8)). Results of computational inferences, including the prediction of metabolic pathways from KEGG (9) or MetaCyc (10), are loaded into the MicroScope data warehouse and made accessible to biologists through a Web user interface (via authenticated or anonymous connections) providing a variety of analytical and visualization tools for comparative analysis and for the exploration of metabolic data. MicroScope supports collaborative expert annotation processes through the use of specific curation tools and graphical interfaces; it helps to develop hypotheses about specific genomes or sets of genes to be experimentally tested (examples are given in (6)).

Here, we report a number of significant new developments in MicroScope over the last three years, including (i) improvements of the user interface of the genome browser and the gene editor and the design of new genome selectors (ii) the integration of new tools for functional annotation of genes and the characterization of genomic regions and (iii) comparative genomic functionalities based on pangenome graphs. Next, we provide an overview of MicroScope data and users. We conclude with some perspectives about new functionalities that will be integrated in the platform.

## IMPROVEMENTS OF THE USER INTERFACE

MicroScope users can query the data and perform diverse comparative analyses through the MaGe web interface (4). Although most of the user interface did not change compared to what has been previously described (6,7), several significant improvements have been made to ease navigation, data exploration and more generally, the use of the platform.

### Genome selection

**Figure 1.**
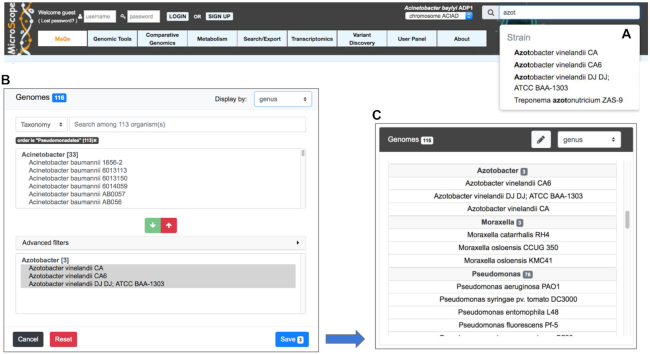
Genome Selectors of the MicroScope platform. Two new widgets for the selection of sequences or genomes are available. The ‘simple selector’ is shown in panel (**A**). It is a search suggest drop-down list that allows users to quickly select the genome of interest while the species or strain name is being typed. In panel (**B**), the ‘advanced selector’ allows users to select multiple sequences or genomes by applying filters based on the taxonomy, strain names or identifiers of MICGCs. It is made up of a pre-selection area that lists all matching entries according to the filters and a selection area to refine the final list of sequences to save using arrow buttons to add (green button) or remove (red button) entries. Optional additional filters (‘Advanced filters’ menu) can be used to remove entries. The saved list is then displayed according to the desired taxonomic level (genus level in panel **C**).

With the increasing number of very diverse prokaryotic genomes being integrated in MicroScope, two new widgets for the selection of sequences (i.e. specific replicons) or genomes (i.e. all the replicons of a genome) have recently been developed to ease this task. The ‘simple selector’ is a search suggest drop-down list for quickly retrieving the genome of interest while the species or strain name is being typed. This text box search is available in the upper right corner of all web pages requiring the selection of a reference genome (Figure 1A). The ‘advanced selector’ is designed to allow users to efficiently select multiple sequences or genomes by applying filters based on the taxonomy, strain names or identifiers of MicroScope Genome Clusters (MICGC, see ‘Toward comparative pangenomics’ section) (Figure 1B). This selection is done in two steps with a pre-selection area that lists all matching entries according to the filters and a selection area to refine the final list of sequences using arrow buttons to add or remove entries using optional additional filters (‘Advanced filters’ menu). The saved list is then displayed according to the desired taxonomic level (Figure 1C). This selector is available on many pages of the platform such as the ‘Gene Phyloprofile’ and the ‘Search by keywords’ tools and all functionalities of the ‘Metabolism’ menu.

### Genome browser

**Figure 2.**
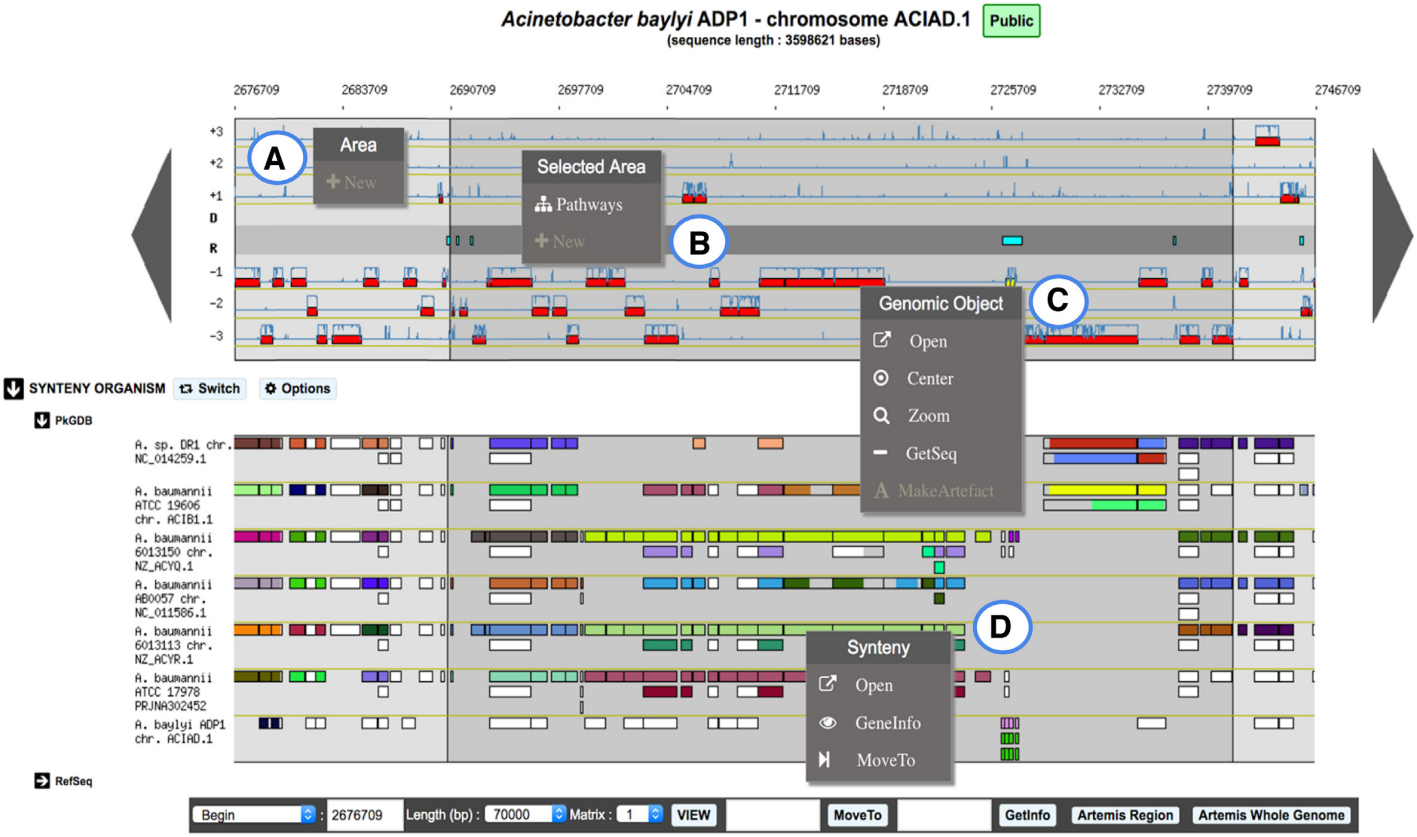
Overview of MicroScope Genome Browser. A 70-kb chromosomal segment from *Acinetobacter baylyi* ADP1, starting at position 2676709, is represented on this graphical map of the MicroScope Genome browser. Annotated CDSs are represented in the six reading frames of the sequence by red rectangles, and coding prediction curves (blue curves) are superimposed on the predicted CDSs. The central part of the viewer, colored in gray, separates the reverse strand of the DNA sequence from the direct one. It displays repeat regions as well as non-coding genomic objects (e.g. tRNA, rRNA, misc_RNA) according to their strand. The synteny maps, calculated on a set of selected genomes, are displayed below the genome viewer (here on seven genomes from MicroScope PkGDB database). New contextual menus in the genome browser allow users: (**A**) to create a new genomic object; (**B**) to list the KEGG metabolic pathways for which enzymes are encoded in a highlighted region; (**C**) to access shortcuts to perform different actions on a specific genomic object: open the gene editor, center or zoom the view around this object, get its nucleic and protein sequences or annotate it as an artefact; (**D**) to explore synteny conservation in other species: open the synteny viewer, get gene information on a homologous gene, move the genome browser to the corresponding region of the compared genome.

The genome browser has been updated to ease the visualization of information on genomic objects. In the cartographic map representing the portion of the genome sequence being analyzed (70 kb of *Acinetobacter baylyi* ADP1 in Figure 2), the central part is now colored in gray to better separate the reverse strand of the DNA sequence from the forward one. It contains repeat regions as well as non-coding genomic objects (e.g. tRNA, rRNA, misc_RNA). In addition, a darker background is drawn to indicate the selected object (e.g. when using ‘MoveTo’ action) or genomic region.

Novel contextual menus have been added in the genome browser to ease the navigation and exploration of genomic objects or genomic regions. For instance, it allows users to quickly center or zoom on a selected gene (Figure 2C), to create a new object (Figure 2A) or to list all KEGG metabolic pathways for which enzymes are encoded in the highlighted region (Figure 2B). In the synteny map, the contextual menu of the rectangle tags (which indicate if a homolog exists in the compared genome) provides access to the gene information page of the homolog or allows users to move the genome browser to the corresponding region of the compared genome (Figure 2D).

### Gene annotation editor

The gene editor has also been improved to offer a more accurate form with functionalities for checking inconsistencies. The annotation fields are now dependent on the type of object being considered (e.g. CDS or RNA). The ‘Note’ field is editable and corresponds to the ‘/note’ qualifier in GenBank/ENA files. Two new fields have been added: (i) ‘Additional data’ allows users to store multiple types of data such as: comments, interactions, phenotypes or SMILES formulas (ii) ‘dbxref’ is used to link external database identifiers related to the annotation of the gene.

Before saving any new (or modified) gene annotation, the procedure allowing to detect annotation inconsistencies between the fields of the gene editor has been improved in order to pinpoint conflicts and/or mistakes (see the MicroScope annotation rules here: https://microscope.readthedocs.io/en/v3.13.3/content/mage/info.html#annotation-rules). Furthermore, the ‘Product type’ terms (inspired from the M. Riley's list set-up for the expert annotation of *Escherichia coli* K-12 genes (11)) were simplified by removing all ‘putative…’ entries (e.g. putative regulator, putative enzyme) since both ‘Product’ and ‘Class’ fields can be used to annotate ‘putative’ functions (i.e. Class ‘3’ refers to ‘putative’ functions).

### Genome overview

Following the modification of MicroScope PkGDB database schema, the management of assembly was improved by providing unique identifiers for each version which are linked to a consistent list of replicons, sequences and scaffolds/contigs that compose the assembly. The ‘Genome Overview’ functionality now summarizes information about the different replicons on the same page. Scaffolds/contigs and gene counts, curation status and other metrics are given for each replicon. The taxonomic lineage and the estimation of the genome completion and contamination with CheckM software (12) are also provided.

## NEW TOOLS FOR FUNCTIONAL ANNOTATION

The development of the MicroScope platform has always been driven by adding new functionalities which aim to help microbiologists performing functional annotation and comparative genomics. Since 2017, several new tools for the functional annotation of genes and genomic regions have been integrated in MicroScope. The results of these tools are summarized for each CoDing Sequence (CDS) in the gene editor page and can be queried through the ‘Search by keywords’ functionality. For some of them, a specific web page has been set up. Besides, our automatic annotation procedure has also been improved as described below.

### Update of the automatic annotation procedure

In order to make the most of the spread of high-quality annotations, the MicroScope automatic annotation pipeline has been improved. The procedure triggers at the end of the functional annotation step, and automatically associates a functional annotation to each predicted CDS. It relies on different computational results such as sequence alignments with proteins from Swiss-Prot or selected reference genomes. To capitalize over new developments made in collaboration with the UniProt EMBL-EBI team, we integrated UniFIRE (The UniProt Functional annotation Inference Rule Engine, https://gitlab.ebi.ac.uk/uniprot-public/unifire) in our automatic annotation procedure. UniFIRE is an open-source Java-based framework and tool to apply the UniProt annotation rules on given protein sequences (13). Two sets of rules are provided (i) the SAAS rules (Statistical Automatic Annotation System) which are generated automatically from expertly annotated entries in UniProtKB/Swiss-Prot, and (ii) the UniRules (The Unified Rule) which are devised and tested by experienced curators. In MicroScope, UniFIRE annotation results are available in the gene editor page in UniFIRE SAAS and UniFIRE UniRules datasets.

Besides, in order to enhance the quality of information delivered to the users working on unfinished genome sequence, we have improved the procedure which maps annotated features of the earlier version of the genome assembly to the new one. The ‘Annotation mapping’ functionality now gives more precise information about the mapping between two versions of assembly. During this process, sequence alignments are performed to compare newly predicted genomic objects with those previously annotated. At the end, annotations of the mapped elements are transferred to the new version of the sequence. In addition, a report is provided with correspondences between new genomic object identifiers (i.e. locus_tag) and the previous ones. Sometimes, the correspondences may not have been established automatically as for example in the case of multiple mapping (i.e. several objects on the previous sequence match the same object on the current sequence). To solve these problems, users are invited to manually check a list of potential mappings available in the ‘Annotation mapping’ page.

### New tools for functional annotation of genes

Since 2017, we have extended our annotation pipeline with new methods helping for functional assignation: functional classification of genes using eggNOG, prediction of essential genes and prediction of genes potentially involved in pathogenicity such as resistance genes (resistome) or virulence genes (virulome).

#### EggNOG classification

EggNOG (evolutionary genealogy of genes: Non-supervised Orthologous Groups) is a resource that provides Orthologous Groups (OGs) of proteins at different taxonomic levels, each with integrated and summarized functional annotations (14). EggNOG uses a graph-based unsupervised clustering algorithm extending the methodology of Clusters of OGs (15) to produce genome wide orthology inferences. EggNOG results (obtained with eggNOG-mapper tool) are available in the MicroScope platform providing functional annotations and classification of CDSs, a summary of which is available in the ‘Genomic Tools’ menu.

#### Prediction of essential genes

Delineating a set of essential genomic elements that makes up a living organism helps to understand critical cellular processes sustaining life. A new ‘essential gene’ dataset from the Database of Essential Genes (DEG) (16) has been set up in MicroScope. It replaces the three original datasets of essential genes of the model organisms *Pseudomonas aeruginosa* PAO1, *E. coli* K-12 and *Bacillus subtilis* 168. DEG contains records for bacterial and archaeal essential genes determined by genome-wide screens. The experiments were performed under diverse conditions including those for survival, pathogenesis and antibiotic resistance. The DEG dataset has further been expanded with data from *A. baylyi* ADP1 and *Neisseria meningitidis* 8013, two highly curated genomes in MicroScope (17,18). ‘Essential genes’ results (obtained from amino-acid sequence alignments) are available for each CDS of all genomes integrated into MicroScope.

#### Prediction of resistome

For a given genome, genes potentially involved in resistance to antibiotics are listed in the ‘Resistome’ section of the ‘Comparative Genomics’ menu. This prediction relies on the use of the Comprehensive Antibiotic Resistance Database (CARD). CARD is a manually curated resource containing high quality reference data on the molecular basis of AntiMicrobial Resistance (AMR), with an emphasis on the genes, proteins and mutations involved in AMR (19). It is built upon the Antibiotic Resistance Ontology, a custom built, interconnected and hierarchical controlled vocabulary allowing advanced data sharing and organization. Every genome integrated into MicroScope is scanned using the RGI (Resistance Gene Identifier) software, which is provided with the CARD resource, for prediction of resistome. This software uses different models (CARD Proteins Homologs, CARD Proteins Variants or CARD overexpression) to detect the AMR from protein sequences. In MicroScope, a summary of the CARD results for each genome is available in the ‘Resistome’ section of the ‘Comparative Genomics’ menu.

#### Prediction of virulome

Similarly, genes potentially involved in the virulence of a given organism (e.g. genes coding for bacterial adhesins, colonization factors, protein toxins like hemolysins) are listed in the ‘Virulome’ section of the ‘Comparative Genomics’ menu. The prediction is based on nucleotide and protein BLAST searches against a homemade virulence database. It was built using three datasets of virulence genes: (i) the core dataset from VFDB (setA), which is composed of genes associated with experimentally verified Virulence Factors (VFs) for 53 bacterial species (20) (ii) the VirulenceFinder dataset which includes virulence genes from *Listeria*, *Staphylococcus aureus*, *E. coli/Shigella* and *Enterococcus* (https://cge.cbs.dtu.dk/services/VirulenceFinder) (21) and (iii) a manually curated dataset of reference virulence genes for *E. coli* (Coli_Ref) (22). In order to ease the functional interpretation of the results, the classification from VFDB is attributed to each VF of VirulenceFinder and Coli_Ref as frequently as possible. VFs are divided into four main classes: offensive, defensive, non-specific and regulation of virulence-associated genes. A VF can be involved in many classes. For example, the gene *kpsE* from *E. coli* (that encodes a capsule polysaccharide export inner-membrane protein) is classified as an offensive and a defensive VF. Predictions can be filtered according to the taxonomy of the studied strain (i.e. results must be obtained with VFs of organisms of the same species or genus) or using different alignment identity thresholds.

### New tools for the characterization of genomic regions

**Figure 3.**
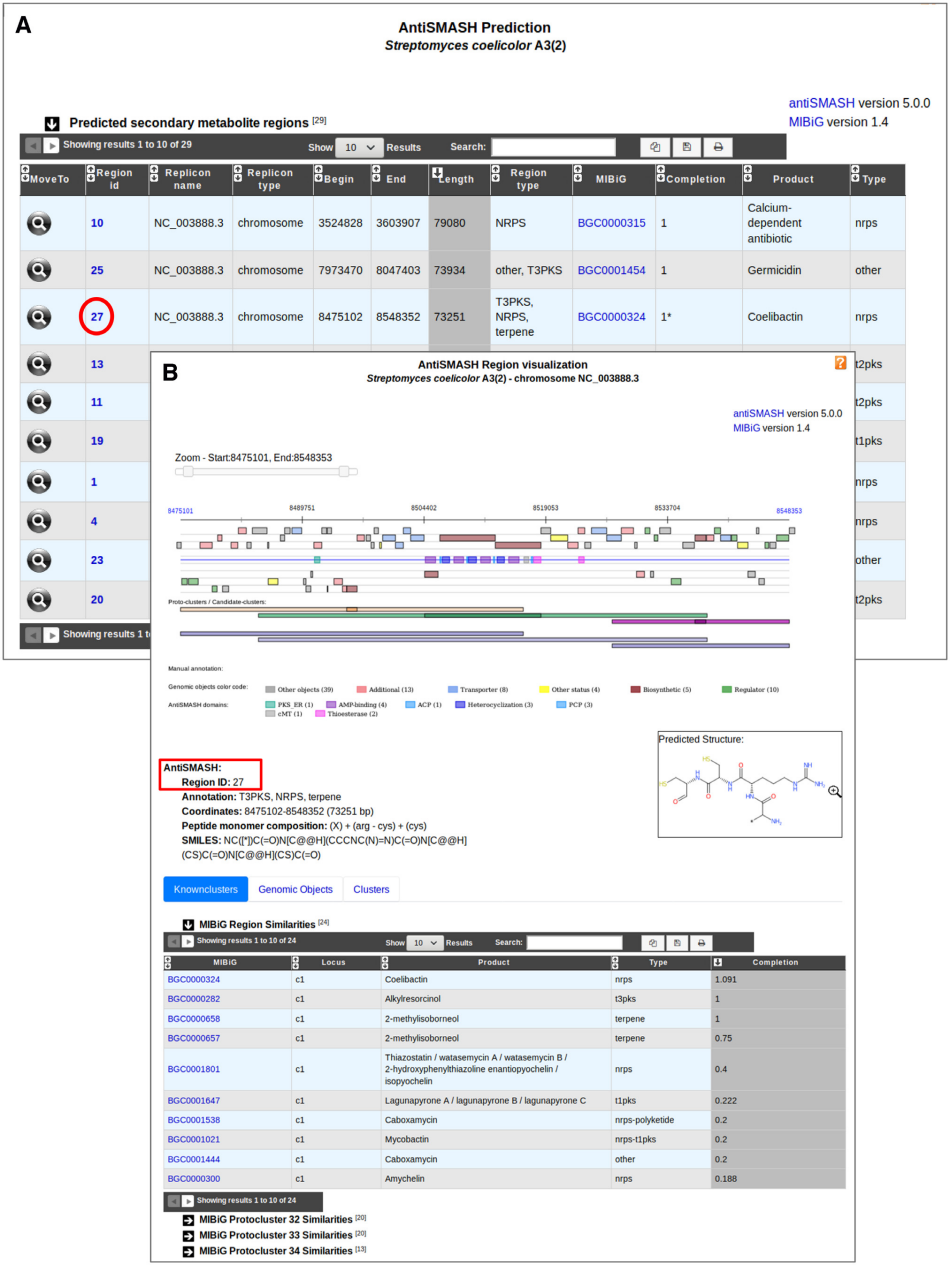
MicroScope ‘Secondary metabolites’ functionality. BGC predictions for an organism can be accessed from the ‘Secondary metabolites’ section of the ‘Metabolism’ menu. It gives access to a table summarizing BGC region predictions for all replicons of a studied organism (panel **A**). Here, we see the list of regions predicted by antiSMASH 5 in *Streptomyces coelicolor* A3(2). The closest known cluster from the MIBiG database is indicated for each prediction if any. All individual predictions can then be explored by clicking on the cluster numbers. An example is shown on the panel **B** for the region number 27. Biosynthetic genes found in the region are colored in brown and their domain composition is indicated as well. Putative transporter and regulator genes are highlighted in blue and green, respectively. Three protoclusters of three different types have been defined in this region: T1PKS (orange), NRPS (green) and terpene (purple). Each of them is associated with a candidate cluster drawn in blue. Basic region characteristics are indicated below the visualization section such as proposed antiSMASH annotation and coordinates. In case of NRPS/PKS BGC type, the peptide monomer composition is indicated with the corresponding chemical structure encoded in SMILES. The ‘MIBiG Region Similarities’ table indicates similarities with known clusters and the completion value. Here, the NRPS (coelibactin), T3PKS (alkylresorcinol) and terpene (2-methylisoborneol) known clusters are retrieved with high completion values.

The ‘Secondary metabolites’ functionality dedicated to secondary metabolite prediction (already described in (7)) has been updated using the version 5 of antiSMASH (antibiotics and Secondary Metabolite Analysis Shell) software, which enables rapid genome-wide identification, annotation and analysis of secondary metabolites (23). The new version detects 52 types of Biosynthetic Gene Clusters (BGCs) covering a large range of known secondary metabolite compound classes that are produced by non-ribosomal peptide synthetases (NRPS), polyketide synthase (PKS) or ribosomally synthesized peptide products (RiPP). The program also offers extended and improved BGC detection and analysis capabilities particularly for regions containing multiple BGCs of one or several types. To help users analyze these regions, the program introduced a new terminology to describe different biological options that lead to BGCs with notably ‘protoclusters’ and ‘candidate clusters’. A protocluster describes a BGC (core enzymes and neighborhoods at both sides of the core) and is associated with one product type. Candidate clusters are designed to allow modeling of hybrid clusters, such as PKS/NRPS hybrids, which combine two or more different biosynthetic classes (as identified in antiSMASH detection rules), or cases where one class is used to biosynthesize a precursor for a second class. Thus, a candidate cluster contains one or several protoclusters. Hence, the MicroScope graphical interface for the visualization of predicted secondary metabolite clusters has been updated to show protoclusters and candidate clusters defined by antiSMASH (Figure 3). This new feature is particularly useful for the analysis of complex regions composed of multiple BGCs. Moreover, corresponding result tables have been split into three sections to describe (i) similarities with known clusters from the MIBiG database, (ii) the list of genomic objects that compose the region and (iii) candidate cluster and protocluster coordinates.

In addition to the update of antiSMASH, the comparative genomics functionalities of the platform have been enriched with new tools dedicated to functional annotation of genomic regions.

First, we have integrated IntegronFinder which is able to detect, with high accuracy, integrons in DNA sequences (24). Integrons are major genetic elements, notorious for their major implication in the spread of antibiotic resistance genes. More generally, integrons are gene-capturing platforms, whose broader evolutionary role remains poorly understood. IntegronFinder uses HMM profiles for the detection of the site-specific integrase, and Covariance Models for the detection of attC sites. In MicroScope, the list of predicted integrons in a selected organism is available through the ‘Integrons’ section of the ‘Comparative Genomics’ menu.

Another tool, called Macromolecular System Finder (MacSyFinder), provides a flexible framework to model the properties of molecular systems (cellular machinery or pathway) including their components, evolutionary associations with other systems and genetic architecture (25). MacSyFinder can detect a broad range of secretion systems: T1SS, T2SS, T3SS, T4SS, T5SS, T6SS, T9SS, Flg, T4P, Tad (26). Moreover, in association with CRISPRfinder, it can detect CRISPR-Cas systems (i.e. Clustered Regularly Interspaced Short Palindromic Repeats arrays and their associated proteins) (27). The list of predicted systems in a selected strain can be accessed through the ‘Macromolecular Systems’ section of the ‘Comparative Genomics’ menu.

## TOWARD COMPARATIVE PANGENOMICS

Before computing pangenomes at the species level, a proper taxonomic assignation of genomes is essential while excluding incomplete and contaminated ones. These pangenomes are then used for comparative genomics purposes with the prediction of regions of genomic plasticity (RGP).

### MicroScope Genome Clusters

**Figure 4.**
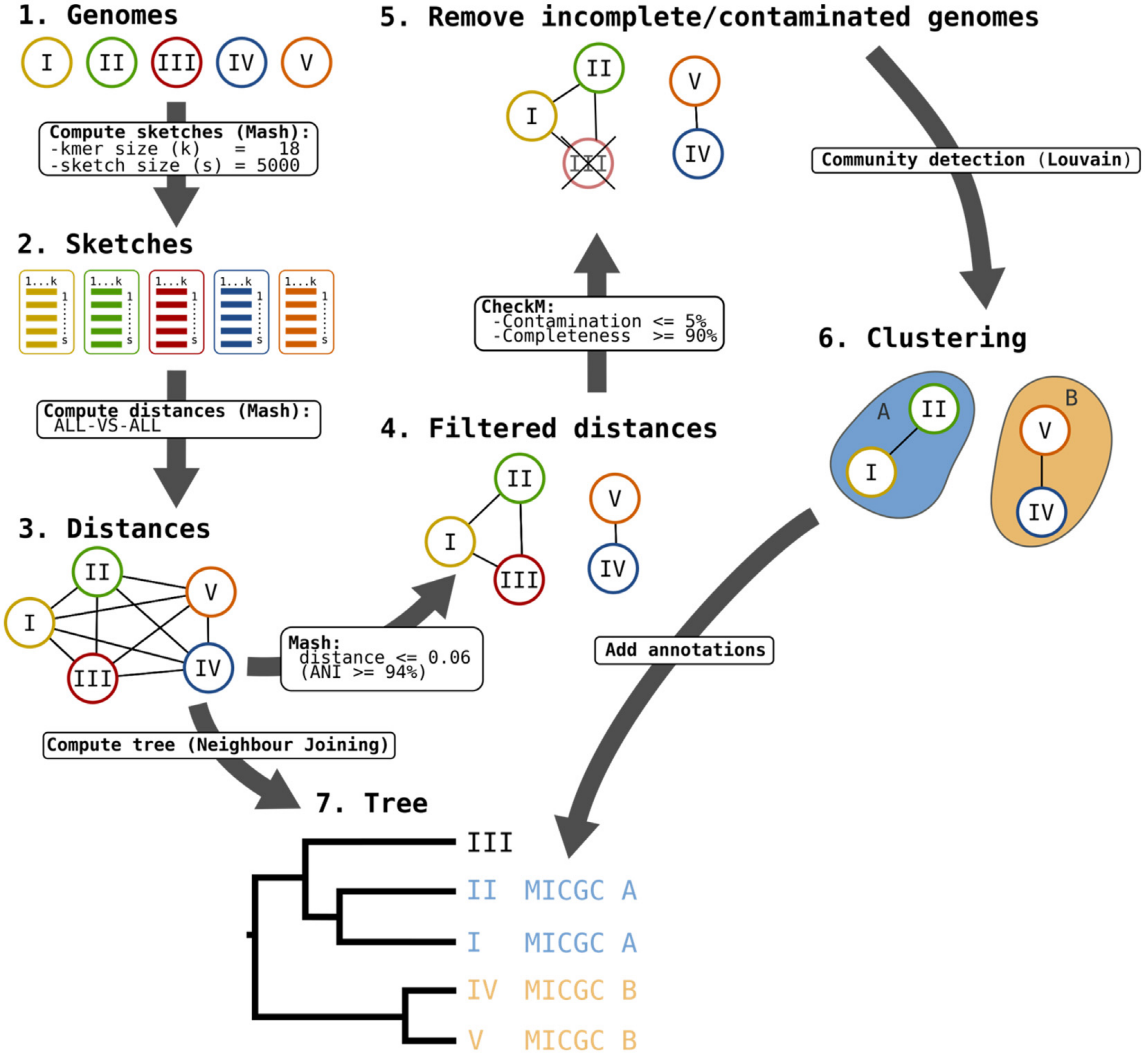
MICGC Workflow. The workflow to compute MICGCs is made of several steps. First genomic distances between all MicroScope genomes are computed using Mash software with kmer and sketch sizes equal to 18 and 5000, respectively. Then, a weighted graph is made from these distances removing edges corresponding to distances higher than 0.06 (which corresponds to a 94% ANI). Using CheckM results, genomes with estimated contamination >5% or completion <90% are removed from the graph. At last, the Louvain community detection method is applied to define species clusters, called MICGCs. Mash genomic distances are also used to compute neighbor joining trees to classify strains (this functionality is available on the ‘Genome Clustering’ page of MicroScope).

We have designed a new workflow to compute MICGCs which aims to group genomes belonging to the same species into clusters (Figure 4). The classification method relies on pairwise genomic distances estimated with the Mash software (28). We consider only distances below or equal to 0.06, which corresponds to a 94% Average Nucleotide Identity (ANI) cutoff that is a usual value to define species (29). Then, the Louvain community detection method (30) is applied to define species clusters, called MICGCs. To avoid bias in the classification, incomplete or contaminated genomes are excluded based on CheckM results (i.e. genomes with estimated contamination >5% or completion <90%).

In addition, a specific web page has been developed to explore MICGCs and is available in the ‘Genome Clustering’ section of the ‘Comparative Genomics’ menu. This interface allows users to select a set of genomes using the ‘Advanced Selector’ (see ‘Improvements of the user interface’ section) and to display a tree that classifies them using a neighbor joining algorithm with Mash genomic distances. The tree can be exported in SVG or Newick format for further analysis.

### Pangenomes and region of genomic plasticity

For each MICGC containing at least 15 genomes, a pangenome is computed using the PPanGGOLiN software (https://github.com/labgem/PPanGGOLiN, Gautreau *et al.*, in preparation). It uses a graph model to represent pangenomes in which nodes and edges represent gene families and genomic neighborhood information, respectively. The pangenome is then partitioned by evaluating, through an Expectation-Maximization (EM) algorithm, the best parameters of a multivariate Bernoulli Mixture Model smoothed using a Markov Random Field. This approach takes into account both graph topology and occurrences of genes to classify gene families into three partitions: (i) persistent genome, equivalent to a relaxed core genome (genes conserved in almost all genomes) (ii) shell genome corresponding to moderately conserved genes potentially associated with environmental adaptation capabilities (iii) cloud genome, genes found at a low frequency. MICFAM (MICroScope FAMilies) homologous gene families are used to construct the pangenome graphs. These families gather protein-encoding genes sharing at least 80% of amino-acid identity and 80% of alignment coverage (7).

Using PPanGGOLiN results, a new functionality to search for potentially horizontally transferred genes gathered in RGP, called PanRGP, has been developed. Based on the projection of the Partitioned Pangenome Graph on a given genome, the PanRGP method defines a RGP as a set of consecutive genes that are members of the shell or cloud genomes. PanRGP applies a score-based algorithm to predict RGPs (>3kb) that correspond to variable regions of the pangenome (Bazin *et al.*, in preparation).

**Figure 5.**
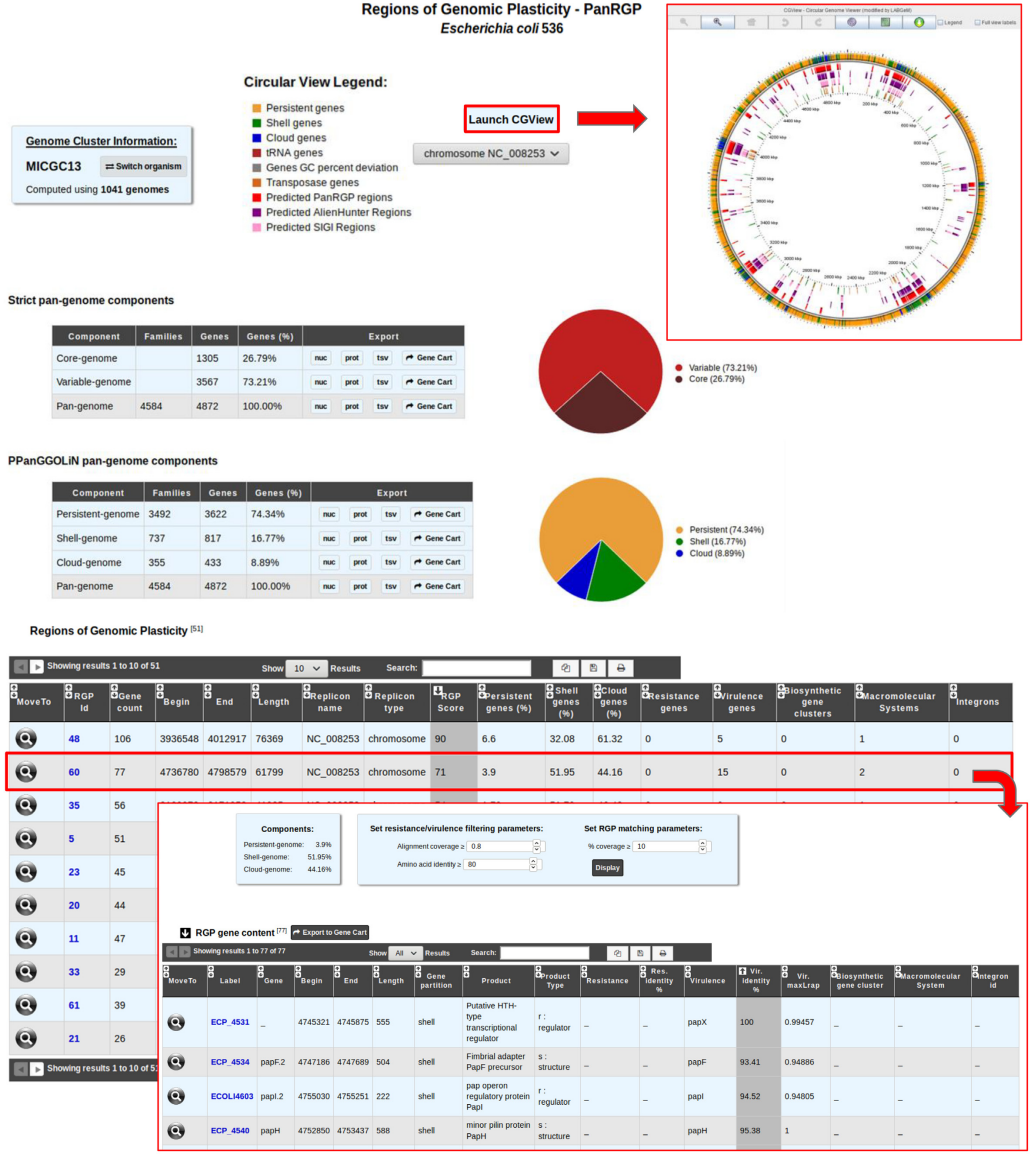
Detection of RGP with PanRGP tool. RGP predictions for a genome can be accessed from the ‘Pan-genome RGPs’ section of the ‘Comparative Genomics’ menu (here for *Escherichia coli* 536). In the genome cluster information panel, the number of genomes of the same MICGC (i.e. species) that were used to compute the pangenome with PPanGGOLiN is indicated. Users may switch to the predictions for another strain using ‘Switch Organism’ button. The ‘Strict pan-genome components’ table represents a summary of the exact core/variable analysis whereas the ‘PPanGGOLiN pan-genome components’ table gives the number of genes and MICFAM families for each PPanGGOLiN partition. Users can extract all this data in fasta files (nucleic and protein), tab-separated values (tsv) files containing the annotations or in a gene cart for further analysis. By clicking on the ‘Launch CGView’ button, it is possible to browse the genes along the genome in a circular representation based on CGView with information about their PPanGGOLiN partition and the RGP locations. The table ‘RGP’ lists all predicted RGPs with a summary of the number of genes involved in antibiotic resistance, virulence, biosynthetic clusters, macromolecular systems and integrons. By clicking on a RGP identifier, a page provides a detailed list of the genes within the selected RGP and a list of similar RGPs in other strains (not shown in the figure).

PanRGP results are available in the ‘Pangenome RGPs’ section of the ‘Comparative Genomics’ menu. As depicted in Figure 5, strict pangenome components (core and variable) as well as PPanGGOLiN pangenome components (persistent, shell and cloud) are computed. PPanGGOLiN partitions and RGP predictions along the genome can also be displayed in a circular graphical representation (31). The ‘RGP’ table gives an overview of all the RGPs predicted by the PanRGP method. To highlight potential genomic islands, RGP results are cross-linked with different functional annotation methods for the prediction of AMR and virulence genes, macromolecular systems, BGCs and integrons. Each RGP can further be analyzed using a dedicated web page that gives their gene content and a list of similar RGPs that were detected in other strains of the same MICGC (i.e. the ‘Matching RGPs within Genome Cluster’ table in which the percentage of shared gene families corresponds to the number of MICFAM families present in the RGP of the compared strain).

## MICROSCOPE USERS AND DATA

Integration and analysis of genomic data into MicroScope, service continuity and data conservation (backup) are currently provided free-of-charge for the worldwide community of microbiologists. The service is mainly used for the annotation of newly sequenced genomes or public prokaryotic genomes and for RNA-seq data analysis. MicroScope user accounts are mostly created during the process of data submission. Since the last update (7), our user community has increased by 65%, with more than 4500 accounts; it remains largely international with only 35% of registered users inside France. During the MicroScope training courses organized by our team, attendees learn how to query and analyze their data, and how to improve the automatic functional annotations. Indeed, since the last update, about 80 people have been trained (during nine basic and advanced MicroScope training courses).

On average per month, we count 370 active accounts (i.e. the user logged in at least once in the month) and 2000 authentications among about 1500 monthly unique visitors. With the huge number of prokaryotic genomes being sequenced today, the time-consuming task of expert annotation is becoming relatively exceptional: biologists focus their annotations on some proteins/functions of interest. However, it is interesting to note that about 132 genomes integrated in MicroScope are near completely curated (≥80% of the genes were expertly annotated), and almost 300 additional genomes got ≥100 curated genes.

In spring 2018, we have organized the first MicroScope Open Days in Paris, which gathered about 80 participants; among them, 8 international invited speakers have shared their experience on using MicroScope for their research projects. Six live demos were presented to highlight recent developments of the platform (i.e. transcriptomics tools, new comparative genomics tools, the Genome Clusters functionality, curation of metabolic pathways, etc.). We also got valuable feedback for future developments of the platform.

Since 2 years, an average of 160 genomes per month are requested for integration in the platform (this includes bins/MAGs from metagenomic samples). The MicroScope resource contains today data for >11 800 genomes of which ∼4400 are publicly available (38%). At the request of the project coordinators, sequences and annotation data are prepared for submission to INSDC databanks; they can also easily be downloaded via the web interface (‘Search/Export→Download Data’ functionality). Two export modes are implemented into MicroScope: (i) the ‘Replicon mode’ to retrieve various data for only one replicon (i.e. chromosomes, plasmids or the whole contigs if the genome is not finished) (ii) the ‘Organism mode’ to download genomic annotation files (EMBL, GenBank, Fasta, GFF3 file formats) in batches of up to 20 genomes. In both modes, genomic data can be split by contig or scaffold entries. At last, MicroScope is one of the resources listed in the catalog of the ELIXIR bioinformatics infrastructure (https://elixir-europe.org/services). In this context, we implemented an export of MicroScope data based on semantic web technology using the Resource Description Framework (RDF). The functionality is available in the ‘Organism mode’ of the export page from which users can download RDF files in Turtle format. These files contain all genomic and metabolic information dynamically extracted from the MicroScope PkGDB database. By defining shared semantic standards, RDF is a way to integrate MicroScope data with other complementary databases such as UniProt (13) and Rhea (32) and to support cross-resource queries using a SPARQL endpoint.

## CONCLUSION

During these last 3 years, we have made an important effort in the enhancement of the user interface and in the integration of new tools for functional annotation. In order to continuously improve the automatic annotation of genes, we intend to work on a system for the propagation of annotations at the level of homologous gene families (i.e. MICFAM families of MicroScope). This system would allow the integration of annotations from different sources (i.e. expert annotations from MicroScope and Swiss-Prot or predictions from UniFIRE and other tools) and the definition of propagation rules at the family level taking into account the homogeneity of the annotations and setting priorities according to the sources.

We have also achieved significant progress in predicting RGP. We intend to better characterize these regions. First, we plan to integrate additional tools to predict genomic regions such as phages. Second, we are currently working on an extension of PanRGP to predict modules within RGPs. These modules correspond to groups of genes that are co-localized and co-occurrent in the pangenome, the conservation of which would indicate a function common to these genes as illustrated by Ogier *et al.* (33).

At last, progress has been made in the consensus representation of thousands of genomes of a species through the pangenome graph model of PPanGGOLiN. The next step will be to use this representation for comparative genomics in MicroScope such as the detection of conserved synteny between a genome and a pangenome or directly between pangenomes.

## DATA AVAILABILITY

MicroScope is available at the following address: https://www.genoscope.cns.fr/agc/microscope. MicroScope services and trainings follow the quality management system of LABGeM laboratory (ISO 9001:2015 and NF X50–900:2016 standards).

## Notes

Present address: Rémi Planel, Hub de Bioinformatique et Biostatistique - Département Biologie Computationnelle, Institut Pasteur, USR 3756 CNRS, Paris, France.

Present address: Valentin Sabatet, Institut Curie, PSL Research University, Centre de Recherche, Laboratoire de Spectrométrie de Masse Protéomique, Paris, France.
